# Chemical composition, and antibacterial and antioxidant activities of essential oils from Laggera tomentosa Sch. Bip. ex Oliv. et Hiern (Asteraceae)

**DOI:** 10.3906/kim-2004-50

**Published:** 2020-12-16

**Authors:** Tokuma GETAHUN, Vinit SHARMA, Deepak KUMAR, Neeraj GUPTA

**Affiliations:** 1 Advance School of Chemical Sciences, Shoolini University, Bajhol, HP India; 2 School of Pharmacy, Faculty of Pharmaceutical Sciences, Shoolini University, Bajhol, HP India; 3 Department of Chemistry and Chemical Sciences, Central University of Himachal Pradesh, Kangra, HP India

**Keywords:** *Laggera tomentosa*, essential oils, 2, 5-dimethoxy- *p* -cymene, thymol methyl ether, antibacterial and antioxidant activities

## Abstract

*Laggera tomentosa* Sch. Bip. ex Oliv. et Hiern (Asteraceae), an endemic Ethiopian medicinal plant, is traditionally used to treat various ailments. Previously, the chemical constituents of the essential oil (EO) of its leaves and inflorescence were documented. However, no data about the chemical compositions of other parts of the EOs of the plant have been reported to date. Moreover, there are no previous biological activity reports on any parts of the EOs of this plant. Thus, in this study, the EOs were isolated from the stem bark and roots of this plant by hydrodistillation and analyzed using gas chromatography-mass spectrometry to identify their components. In addition, antibacterial potentials of the oils were evaluated using the disc diffusion and minimal inhibitory concentration (MIC) methods. 2,2-diphenyl-1-picrylhydrazyl (DPPH) and hydrogen peroxide methods were also employed to assess their antioxidant properties. Oxygenated monoterpenes (71.82% and 77.51%), of which 2,5-dimethoxy- *p* -cymene (57.28% and 64.76%) and thymol methyl ether (9.51% and 8.93%) were identified as major components in the EOs of stem bark and roots of *L. tomentosa* and the oils, were the most potent in the DPPH (IC50, 0.33 ± 1.10 and 0.39 ± 0.97 mg/mL) assay, respectively. Moreover, the EOs demonstrated appreciable activity towards the gram+ ( *S. aureus* and *B. cereus* ) bacteria. Among these oils, the oil of the stem bark showed the greatest activity to the gram+ (MIC = 0.625 mg/mL) bacteria. Therefore, the overall results suggested that the EOs of *L. tomentosa* may be a promising prospect for pharmaceutical, food, and other industrial applications.

## 1. Introduction

The genus *Laggera* Sch. Bip. ex Benth. & Hook., belonging to the Asteraceae (Compositae) plant family, has about 20 species, distributed mainly in sub-Saharan Africa and southeastern Asia [1]. Most of its species are often used in folk and traditional medicines for the treatment of inflammation, jaundice, leukemia, bronchitis, removing phlegm, and bacterial diseases, and their leaves, as well as aerial parts, have been reported to have antiinflammatory, antibacterial, antiviral, antioxidant, hepatoprotective, insecticidal, antifungal, anthelmintic, sedative, antituberculosis, and antidiarrheal properties [2]. The essential oils (EOs) of *Laggera* species have also been reported to exhibit antibacterial, antifungal, antioxidant, larvicidal, and insecticidal activities [3–5]. To date, the chemical constituents of the extracts and/or EOs of only 8 plants of the genus *Laggera* have been reported from 13 countries [2]. However, many bioactive compounds with many-sided activities have been identified from these plants, which has drawn researchers to further investigate *Laggera* species [1,2]. The genus is rich in flavonoids, cyclitols, monoterpenes, and sesquiterpenes (eremophilanes and eudesmanes). Flavones, fatty acids, and their derivatives, sterols (stigmasterol and β-sitosterol), phenolic acids like 2-hydroxybenzoic acid, and phytotoxic thymol derivatives, such as 3-hydroxythymoquinone and 5-acetoxy-2-hydroxythymol, have also been reported from extracts of the plants of the genus *Laggera* [1,2,6,7]. 2,5-dimethoxy- *p* -cymene, α-humulene, β-caryophyllene, *γ* -eudesmol, 10- *epi* - *γ* -eudesmol, and juniper camphor are the most frequent chemical compounds in the EOs of these plants, and the former (oxygenated monoterpene) is the most abundant, as well as the most dominant constituent of many of the EOs [2].

*Laggera tomentosa* Sch. Bip. ex Oliv. et Hiern, an annual fragrant subshrub or bushy herb, is an endemic medicinal plant of Ethiopia. Its leaves are used to treat various diseases, including the common cold, cough, flu, rabies, leech infestation, dysentery, and febrile illness and headaches, while the aerial parts are used for the treatment of toothache, swelling, and ringworm, and the roots are used for the treatment of evil eye [2]. The plant is also reported to be used against migraine, as a fumigant, as a treatment for stomachache, and for cleansing milk containers [8]. It is also used to treat skin infections and external parasites [9]. Previously, the chemical constituents of the EO of the leaves and inflorescence of *L. tomentosa* were documented [10]. However, to the best of our knowledge, and according to a literature survey, no data about the chemical compositions of other parts of the EOs of the plant have been reported to date. Moreover, there are no previous biological activity reports on any parts of the EOs of this plant. Therefore, in this paper, various chemical constituents of the stem bark and roots of *L. tomentosa* EOs were isolated and analyzed, respectively, by hydrodistillation and gas chromatography-mass spectrometry (GC-MS). Moreover, the antioxidant and antibacterial potentials of the oils, which may be useful in foods, pharmaceuticals, and other industries, were also assessed and reported.

## 2. Experimental

### 2.1. Plant materials

Fresh stem bark and roots of *L. tomentosa* were collected from Daletti, about 26 km southwest of Addis Ababa, Ethiopia, near the town of Alemgena, in April 2019. The plant materials were authenticated by Professor Legesse Negash and a voucher specimen (No. 00L1) was deposited at the Ethiopian National Herbarium of the Addis Ababa University.

### 2.2. Extraction and isolation of the essential oils

EOs were obtained from the dried and powdered samples (150 g each) of the stem bark and roots of *L. tomentosa* by hydrodistillation using a Clevenger-type apparatus for about 3 h. The obtained oils were separated completely from the condensed water using a separation funnel to afford 0.17% w/w of light yellow and 0.12% w/w of pale yellow EOs from the stem bark and roots of *L. tomentosa* , respectively. The separated oils were then dried by anhydrous Na2SO4 and kept in sterilized dark glass bottles in the refrigerator at 4 °C prior until the analyses.

### 2.3. GC-MS analysis of the oils

Samples of each of the EOs, extracted as described above, were diluted in n-hexane (1:10) and analyzed using GC-MS (Thermo Fisher Scientific Inc., Waltham, MA, USA), which was equipped with a DB-5 (30 m × 0.25 mm i.d., 0.25-µm film thickness) capillary separation column. GC/MS operating conditions comprised the following: oven temperature, from 50 to 250 °C, at increments of 5 °C/min, and held for 5 min; injector temperature, 220 °C ; transfer line, 250 °C; carrier gas, He at 1.0 mL/min constant flow rate; injection volume of the sample, 1 µL; split ratio, 1:20. Ionization of the components of the EO samples was performed in electron impact (70 eV) mode, at an m/z range of 40–450. The identity of the components of the EOs was achieved by visual interpretation and comparing their retention indices (RIs) relative to the n-alkanes of the C7–C30 and mass (m/z) spectra with those from the literature [11–13], and NIST and Wiley libraries.

### 2.4. Antioxidant activities

Antioxidant activities of the EOs of the stem bark and roots of *L. tomentosa* were determined using 2,2-diphenyl-1-picrylhydrazyl (DPPH) and hydrogen peroxide (H2O2) assays at final concentrations within the range of 0.03 to 1.0 mg/mL. Ascorbic acid (AA) was used as a positive control in both assays.

#### 2.4.1. 2,2-diphenyl-1-picrylhydrazyl assay

The method described by Lesjak et al. [14], with some modifications, was used to test antioxidant activity of the EOs by DPPH. Three milliliters of standard solution of each of the concentrations, from 0.03 to 1.0 mg/mL, was mixed with 1.0 mL of 90 µM of DPPH solution in methanol (MeOH) to make the test solutions. AA was prepared in same way as the test samples. A mixture of 3 mL of MeOH and 1 mL of DPPH solution was used as the negative control. Each assay was performed 3 times and the prepared samples were incubated in the dark at 37 °C for about 30 min. Next, the absorbance for each was determined at a wavelength of 515 nm using a spectrophotometer. The antioxidant activity of all of the oils was expressed as the percentage of DPPH radical scavenging and the 50% inhibitory concentration (IC50) (mg/mL).

#### 2.4.2. Hydrogen peroxide assay

The H2O2 scavenging activity of the EOs was investigated 3 times using the method clearly described by Gülçin [15]. The concentrations, from 0.03 to 1.0 mg/mL, of each of the oils and AA in deionized water were dissolved in 3.4 mL of 0.10 M phosphate buffer at a pH of 7.4 and mixed with H2O2 (40 mM, 0.60 mL) solution. After few minutes, the absorbance of the mixture was determined at 230 nm using a UV/Vis spectrophotometer. The negative control was prepared by replacing the test samples with distilled water. The antioxidant activity of all of test samples was expressed as the percentage of inhibition of the H2O2 and as the IC50 (mg/mL).

### 2.5. Bactericidal activities

Disc diffusion analysis was performed to assess the antibacterial activity of the oils against 2 gram+ ( *Staphylococcus aureus* ATCC 25923 and *Bacillus cereus* ATCC 10876) and 2 gram– ( *Klebsiella pneumoniae* ATCC 70063 and *Escherichia coli* ATCC 25922) bacteria at different (0.10, 0.25, and 0.50 mg/mL) concentrations using the method described by Luo et al. [13], with slight modifications. For this method, 6-mm-diameter sterilized Whatman No. 1 filter paper disks were saturated with different concentrations of the EOs of the stem bark and roots of *L. tomentosa* , and placed on nutrient agar plates. The plates were preinoculated with each of the test bacterial strains in suspension (108 CFU/mL) of bacteria and then incubated for about 24 h at 37 °C. After incubation, the diameters of their inhibition zones (DIZs) were measured in millimeters. The minimal inhibitory concentration (MIC) was assessed using the broth dilution method [16], with some modifications. The EOs were dissolved in 10% dimethyl sulfoxide and added to Mueller Hinton broth for the growth of the bacteria. All of the test samples were prepared with the final (10 to 0.015 mg/mL) concentrations and distributed in 96-well microplates. In each test, the bacterial inocula were added into each of the growth control wells. The MIC of each of the samples was then recorded as the lowest concentration of the EOs that inhibited bacterial growth, after incubation for 18–24 h at 37 °C. The antibiotic gentamicin was used as a control (positive) against the selected bacterial strains.

### 2.6. Statistical analysis

All of the experimental results were expressed as the mean ± standard deviation (SD) of 3 repeated trials. A comparison of the group means and the difference between the groups (P < 0.05) were verified using the Student’s t-test.

## 3. Results and discussion

### 3.1. Composition of the essential oils

EOs and their constituents are widely used in the food, pharmaceutical, and cosmetics industries [17]. The chemical names of the identified constituents of the EOs isolated from the stem bark and roots of *L. tomentosa* , together with their RIs and percentages, are given in Table 1, where the compounds have been listed in the order of elution on the DB-5 column used. A total of 76 volatile components were detected in the EO of the *L. tomentosa* stem bark, which represented about 91.84% of the oil extracted. The dominant compounds were 2,5-dimethoxy- *p* -cymene (57.28%), thymol methyl ether (9.51%), humulene epoxide II (5.96%), and orcinyl tiglate (3.78%). For the *L. tomentosa* roots, 55 compounds, representing about 96.51% of the oil, were identified, with 2,5-dimethoxy- *p* -cymene (64.76%), thymol methyl ether (8.93%), palmitic acid (5.18%), and α-humulene (2.34%) being the most dominant (Table 1, Figure).

**Table 1:** Chemical composition of the EOs from the stem barks and roots of L. tomentosa.

No.	RIa	Constituents	Area%	
			SBEO ^b^	REO ^c^
		Oxygenated monoterpenes	71.82	77.51
1	1104	Linalool	0.09	0.05
2	1126	cis-2-menthenol	0.05	tr
3	1145	trans-2-menthenol	tr	tr
4	1149	trans-verbenol	0.73	0.09
5	1162	cis-chrysanthenol	0.08	0.23
6	1183	4-terpineol	0.06	tr
7	1192	p-cymen-8-ol	0.07	0.05
8	1199	cis-piperitol	tr	
9	1212	trans-piperitol	tr	tr
10	1229	8,9-dehydrothymol	tr	
11	1231	cis-carveol	0.08	tr
12	1234	cis-geraniol	0.05	tr
13	1243	Thymol methyl ether	9.51	8.93
14	1251	Carvacrol methyl ether	tr	tr
15	1258	8,9-dehydrothymol methyl ether	tr	
16	1291	Thymol	0.09	1.58
17	1297	Carvacrol	1.74	0.07
18	1308	6-ethyl-3,4-dimethylphenol	0.11	tr
19	1316	4-terpinenyl acetate	tr	tr
20	1327	6-hydroxycarvotanacetone	0.61	0.88
21	1333	trans-piperitol acetate	tr	tr
22	1339	Piperitenone	0.05	0.14
23	1358	Eugenol	0.20	0.31
24	1405	3-(2-methoxyphenyl)-3-pentanol	tr	
25	1427	1,4-dimethoxy-2-tert-butylbenzene	tr	
26	1434	2,5-dimethoxy-p-cymene	57.28	64.76
27	1442	cis-geranylacetone	0.06	0.26
28	1447	2,4-diisopropylphenol	0.11	tr
29	1453	Δ8,9-dehydro-4-hydroxythymol dimethyl ether	0.42	0.16
30	1461	trans-geranylacetone	tr	
31	1501	3-tert-butyl-4-methoxyphenol	0.24	
32	1558	Methyl-3-(2,6,6-trimethyl-1-cyclohexen-1-yl)propionate	tr	
33	1576	Neryl (S)-2-methylbutanoate	0.19	
		Oxygenated sesquiterpenes	10.28	7.84
34	1536	Nerolidol	tr	0.12
35	1563	7-epi-cis-sesquisabinene hydrate	tr	
36	1584	Humulene epoxide I	0.09	
37	1587	(-)-Spathulenol	0.18	0.06
38	1593	Caryophyllene oxide	0.35	1.89
39	1623	Humulene epoxide II	5.96	1.12
40	1641	Caryophylla-4(12),8(13)-dien-5α-ol	0.13	0.96
41	1648	Isoaromadendrene epoxide	2.02	tr
42	1662	Hinesol	tr	0.62
43	1672	Agarospirol	0.99	1.37
44	1684	-α-bisabolol	tr	
45	1702	Ledene oxide	0.14	
46	1710	Germacra-4(15),5,10(14)-trien-1α-ol	0.12	0.43
47	1745	10-epi-γ-eudesmol acetate	0.11	1.06
48	1753	α-sinensal	0.19	0.21
		Sesquiterpene hydrocarbons	<1.5	4.47
49	1849	Hexahydrofarnesyl acetone	tr	tr
50	1424	β-caryophyllene		1.17
51	1451	α-humulene	1.23	2.34
52	1464	9-epi-β-caryophyllene	tr	
53	1486	Germacrene D		0.96
54	1491	α-selinene	0.08	
55	1514	δ-cadinene	0.05	tr
		Alkanes	<0.5	<0.5
56	1400	Tetradecane	0.08	tr
57	2000	Eicosane	tr	0.09
58	2100	Heneicosane	tr	tr
59	2200	Docosane	0.09	0.07
60	2300	Tricosane	0.07	tr
61	2400	Tetracosane	tr	tr
		Fatty acids	0.08	5.32
62	1279	Pelargonic acid	0.08	
63	1569	Lauric acid		0.14
64	1966	Palmitic acid		5.18
		Angelates and tiglates	5.99	<0.15
65	1667	Thymyltig late	1.84	tr
66	1759	Orcinylange late	0.37	tr
67	1825	Orcinyltig late	3.78	
68	1844	6-methoxy thymyl tiglate	tr	
		n-alkyl alcohols	<0.2	<0.2
69	1074	Caprylic alcohol	0.08	tr
70	1174	Pelargonic alcohol	0.05	
71	1274	Capric alcohol	tr	0.07
72	1474	Lauryl alcohol		tr
		Others	1.47	0.52
73	794	2-hexanone	tr	
74	946	3-hydroperoxyhexane	tr	
75	972	1,3-dithiolane	tr	
76	1038	α-hydroxytoluene	tr	
77	1187	o-xylenol	0.08	tr
78	1492	2,6-dimethoxyacetophenone	0.37	
79	1517	2,4-di-tert-butylphenol	tr	0.38
80	1571	2,4,6-trimethoxyacetophenone	0.51	
81	1655	Asarone	0.59	0.14
		Unidentified compounds	5.77	
82	1766	Unidentified (I)	0.97	
83	1796	Unidentified (II)	4.80	

**Figure:**
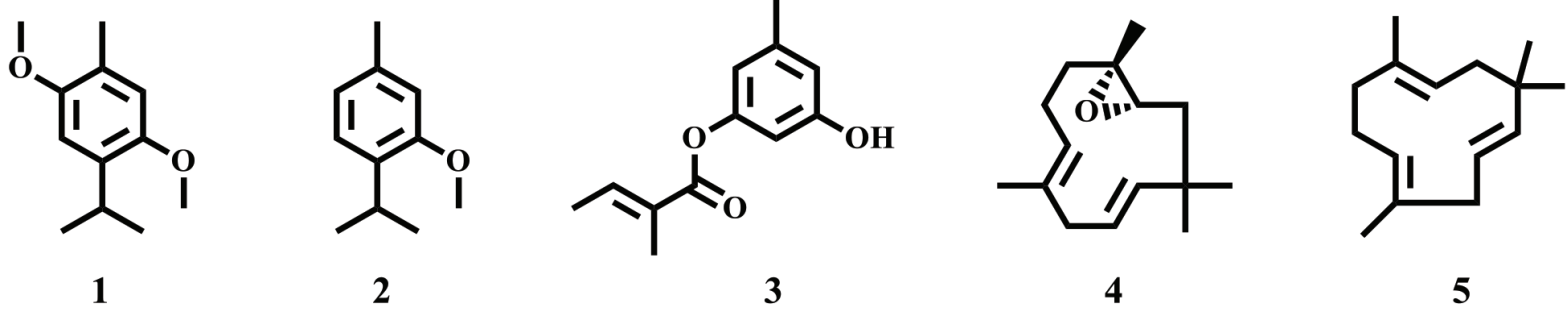
Structures of the major components from the EOs of L. tomentosa. 2,5-dimethoxy-p-cymene (1), thymol methyl ether (2), orcinyl tiglate (3), humulene epoxide II (4), and α-humulene (5).

The dominant compounds of both oils showed similarities, since both were from different parts of the same plant. However, they had also several uncommon compounds. It has also been reported that the chemical constituents of EOs differ in each species or subspecies [18]. As clearly shown in Table 1, both the stem bark and root oils were dominated mostly by oxygen-containing monoterpene compounds (71.82% and 77.51%), the major ones being phenolic ether thymyl derivatives 2,5-dimethoxy- *p* -cymene and thymol methyl ether (total 66.79% and 73.69%), respectively. Oxygenated sesquiterpenes (10.28%), of which humulene epoxide II (5.96%) also occurred in a substantial amount, were detected in the stem bark oil. Sesquiterpene hydrocarbons, such as α-humulene (2.34%), *β* -caryophyllene (1.17%), and germacrene D (0.96%), constituted about 4.47% of the oil that were identified in the root EO, of which the last 2 volatile compounds were absent in the stem bark EO. Relatively high concentrations of fatty acids (5.32%), of which 5.18% was palmitic acid, were also observed as constituents in the root oil. However, monoterpene hydrocarbon compounds were not detected in the 2 oils, as it may have been oxidized to form the dominant oxygenated monoterpenes.

Two compounds ( **I** and **II** ), of which **II** , at high concentration (4.80%, Table 1), in the EO of the stem bark of *L. tomentosa* , were not identified. The mass spectra of the 2 compounds were similar, but their GC retention times (RTs), as well as their RIs, were different. From their mass spectra, the base peak m/z 82 of **I** and m/z 83 of **II** may have been due to the base peaks of the angelates or tiglates. However, due to the lack of RTs, RIs, and mass (m/z) spectra in the literature, the 2 compounds were not identified. Surprisingly, the constituents of both oils (especially their dominant compounds) differed from that of the EO isolated from the leaf and inflorescence of the same plant [10]. The dominant components of this oil were chrysanthenone (57.5%), isochrysanthenone (6.8%), filifolone (5.2%), and (Z)-isogeranic acid (4.5%), which were not identified as major compounds from *L. pterodonta* , *L. alata* , *L. crispata* , *L. decurrens* , *L. aurita* , *L. gracilis* , and *L. oloptera* . These great variations in the compositions of the EOs of the leaf and inflorescence, as well as the roots and stem bark of *L. tomentosa* and their contents, may have been due to environmental conditions, agronomic management, postharvest technology, storage conditions, genetic factors, and analysis conditions [2]. However, the chemical class of this oil and those of the stem bark and roots was similar, as the oil was rich in oxygenated monoterpenes (78%) [10]. When compared with the EO profiles of the other investigated *Laggera* species mentioned above, the oxygenated monoterpene, 2,5-dimethoxy- *p* -cymene, detected as the dominant compound in the present study was most abundant, as well as the most dominant chemical compound of many of these oils [2]. For example, it was the predominant compound of the EOs of the herb (44.2%) [19] and leaves (29.17%) [20] of *L. alata* in Nigeria; the leaves (34.1%) of *L. alata* var. *montana* growing in Cameroon [21]; the whole *L. alata* plant (24.4%) in Kenya [5]; the fresh leaves (28.2%) and winged stems (50.5%) of *L. pterodonta* in Cameroon [22]; the leaves (36.75%) of *L. pterodonta* in China [23]; the leaves (43.3%) of *L. pterodonta* in Côte d’Ivoire [24]; the leaves (27.7%) of *L. aurita* in Burkina-Faso [25]; the leaves (33.4%) of *L. gracilis* , and the roots (67.8%) and stems (36.5%) of *L. oloptera* in Cameroon [21]; and the aerial parts (43.2%) [26] and (32.2%) [27], leaves (22.28%), flowers at bud stage (48.99%), flowers at full bloom/shattering stage (64.01%), stems (62.81%), and roots (75.55%) [28] of *L. crispata* in India. However, the EOs of the studied *Laggera* species showed different chemical classes of compounds [2]. These variations may have been due to the factors mentioned earlier.

### 3.2. Antioxidant activities of the essential oils

Assessed EOs of *L. tomentosa* were able to reduce stable purple DPPH radical to the yellow DPPH-H with IC50 values in the range of 0.33 ± 1.10 to 0.43 ± 1.12 mg/mL (Table 2). As shown in Table 2, the EO of the stem bark of *L. tomentosa* had slightly higher radical scavenging activity (lowest IC50 value, 0.33 ± 1.10 mg/mL) than the root EO (IC50 value, 0.39 ± 0.97). When compared with the H2O2 assay, the EOs exhibited better antioxidant activities in DPPH than that in H2O2 (Table 2). The overall inhibitory activity of the EOs against H2O2 can be presented in the following order: AA (0.14 ± 0.56 mg/mL) > stem barks EO (0.36 ± 1.05 mg/mL) > roots EO (0.43 ± 1.12 mg/mL). However, the oils showed potent antioxidant activities in all of the tested concentrations when compared with AA. This observed activity of the oils was most likely related to their dominant constituents, such as 2,5-dimethoxy- *p* -cymene and thymol methyl ether (oxygenated monoterpenes). Linalool, terpineol, δ-cadinene, and phenolic chemical compounds, such as eugenol, thymol, and carvacrol, which were also present in the oils, may also have had a significant contribution to the antioxidant activity of the oils [29–31].

**Table 2:** Antioxidant effect of standard and EOs from L. tomentosa by DPPH and H2O2 assays.

Assay	L. tomentosa EOs		Standard
	REO	SBEO	AA
DPPH (IC50*, mg/mL)	0.39 ± 0.97	0.33 ± 1.10	0.09 ± 0.89
H2O2 (IC50*, mg/mL)	0.43 ± 1.12	0.36 ± 1.05	0.14 ± 0.56

Values are expressed as the mean ± SD (n = 3); *IC50: 50% inhibitory concentration; DPPH: 2,2-diphenyl-1-picrylhydrazine, H2O2: hydrogen peroxide, AA: ascorbic acid (positive control).

To the best of our knowledge, and according to literature survey, there have been no previous antioxidant activity reports for the EOs and extracts of any parts of *L. tomentosa* . However, among the 20 *Laggera* species, antioxidant activities of the EOs of the aerial parts of only 2 plants, namely *L. decurrens* and *L. aurita* , were reported. For the oils of these plants, good antioxidant activity values that were slightly higher than the current results, were reported [3,32]. At a concentration of 1 mg/mL, the EO of *L. decurrens* , with high content of 3-methoxythymoquinone (28.1%), showed strong antioxidant activity (93.1%), which was comparable to that of AA (96%). However, the EO of *L. aurita* , with a dominant compound of hexadecanoic acid (21.2%), showed moderate antioxidant activity (81.40 ± 1.98% at 4 mg/mL) in the DPPH assay.

### 3.3. Bactericidal activities of the essential oils

Results from the disc diffusion tests for the in vitro antibacterial activity of the EOs of the roots and stem bark of *L. tomentosa* are presented in Table 3. These oils displayed slightly variable activity towards the investigated bacterial strains. As shown in Table 3, the DIZs of the studied oils ranged from 2.79 ± 0.97 to 9.81 ± 0.70 mm for the different (0.10, 0.25, and 0.5 mg/mL) concentrations tested. The gram– pathogen, *E. coli* , was the most resistant, while the gram+ bacterium, *S. aureus* , was the most sensitive microorganism to the EOs. The DIZs of the stem bark EO were 9.81 ± 0.70 and 8.15 ± 1.32 mm, while those of the root EO were 8.47 ± 0.93 and 7.42 ± 1.26 mm for *S. aureus* and *B. cereus* , respectively, at a concentration of 0.5 mg/mL. The results indicated the susceptibility of the gram+ bacteria to the EOs. However, the oils showed no inhibitory activity towards *E. coli* at any of the concentrations and *K. pneumoniae* at 0.10 and 0.25 mg/mL (Table 3). The results of the MIC values (Table 4) also indicated that the oils were more sensitive against gram+ microorganisms than gram– ones, which were similar to the results obtained by the disc diffusion method. The EO of the stem bark showed the greatest activity against the gram+ (MIC = 0.625 mg/mL) bacteria, while that of the roots demonstrated moderate activity (MIC = 1.25 mg/mL) against the same bacterial strains. However, both oils were least active against the gram– (MIC = 2.5 mg/mL) bacteria.

**Table 3:** Antibacterial activity of the investigated EOs of L. tomentosa.

EOs	Concentration(mg/mL)	DIZ (mm)			
		Gram+		Gram–	
		S. aureus	B. cereus	E. coli	K. pneumoniae
REO	0.10	2.92 ± 1.63	2.79 ± 0.97	NI	NI
	0.25	5.33 ± 1.21	5.25 ± 0.88	NI	NI
	0.50	8.47 ± 0.93	7.42 ± 1.26	NI	1.61 ± 1.59
SBEO	0.10	3.45 ± 1.41	2.58 ± 1.13	NI	NI
	0.25	5.84 ± 1.02	5.04 ± 0.94	NI	NI
	0.50	9.81 ± 0.70	8.15 ± 1.32	NI	2.34 ± 1.16

Results are presented as the mean ± SD (n = 3), DIZ: diameter of inhibition zones, NI: no inhibition.

**Table 4:** Minimal inhibitory concentrations (MICs) of the EOs isolated from the stem barks and roots of L. tomentosa.

EOs	MIC (mg/mL)			
	Gram+		Gram–	
	S. aureus	B. cereus	E. coli	K. pneumoniae
REO	1.25	1.25	2.5	2.5
SBEO	0.625	0.625	2.5	2.5

These results were the first antibacterial activity results for *L. tomentosa* EOs. From the overall results, it can be concluded that if the concentration of these oils increases, their inhibitory effect against the bacteria will also increase. The high activity of the oils of *L. tomentosa* (especially the stem bark oil) against the gram+ ( *S. aureus* and *B. cereus* ) bacteria was most likely due to the chemical compounds having antibacterial properties in them. EOs that contain 2,5-dimethoxy- *p* -cymene as a major component of the oil have been reported to have antibacterial properties [33]. Therefore, the bactericidal activity of the *L. tomentosa* oils may have mainly been related to this compound. Thymol methyl ether, which represented 9.51% and 8.93%, respectively, of the oils of the stem bark and roots of *L. tomentosa* , and a major constituent of the stem bark oil, humulene epoxide II (5.96%), may also have had a significant contribution to the antibacterial activity of the oils. Other compounds, including terpineol, δ-cadinene, α-humulene, caryophyllene oxide, eugenol, thymol, carvacrol, linalool, and geraniol, which were also present in the oils, have been reported to have antibacterial activities [29,31,34–36], and may also have collectively had a remarkable contribution to the bactericidal activities of the oils. According to a literature survey, a detailed list of the in vitro bactericidal activities against a broad set of gram+ and gram– bacteria for different EOs of plants of the genus *Laggera* was reported [2,3,32]. The antibacterial activity results of the oils of these plants, and those obtained in the current study, showed variations that may have been due to factors such as the composition and concentration of the EOs, and the type and concentration of the target organisms [37,38], in addition to the factors stated earlier in Section 3.1. However, among the investigated oils of these plants, the EO of *L. decurrens* , which has high 3-methoxythymoquinone (28.1%) content, showed the highest antibacterial activity (MIC = 0.13 mg/mL) against *S. aureus* [32]. The EO of *L. pterodonta* , which has high 2,5-dimethoxy- *p* -cymene (36.75%) content, displayed moderate activity against some gram– bacteria, such as *Enterobacter aerogenes* (MIC = 0.125 mg/mL), *Enterococcus faecalis* (MIC = 0.5 mg/mL), *E. coli* (MIC = 0.25 mg/mL), *Salmonella typhi* (MIC = 0.5 mg/mL), and *Pseudomonas aeruginosa* (MIC = 0.5 mg/mL) [23]. The EO of *L. crispata* , which has a major compound of 2,5-dimethoxy- *p* -cymene (43.2%), also demonstrated moderate antibacterial activity against *Klebsiella pneumoniae* (ZI = 6 mm) and *Staphylococcus aureus* (ZI = 8 mm), but no activity against *E. coli* [26].

## 4. Conclusion

The compositions of the EOs of endemic Ethiopian *L. tomentosa* stem bark and roots were analyzed and their antioxidant and bactericidal activities were investigated for the first time herein. The oxygenated monoterpenes, 2,5-dimethoxy- *p* -cymene, as well as thymol methyl ether, were identified as the dominant compounds of both oils, which differed from the previously reported chemical profile of the EOs obtained from the leaves and inflorescence of the same plant. Furthermore, the 2 oils demonstrated strong DPPH and H2O2 scavenging activities and bactericidal activity against the gram+ ( *S. aureus* and *B. cereus* ) bacteria, which might have been due to their high oxygenated monoterpene content. Thus, these activities suggest that the oils may be a promising prospect for pharmaceutical, food, and other industrial applications.

## References

[ref1] (2007). Chemical constituents of the plants from the genus Laggera. Chemistry and Biodiversity.

[ref2] (2019). The genus Laggera (Asteraceae)–ethnobotanical and ethnopharmacological information, chemical composition as well as biological activities of its essential oils and extracts: a review. Chemistry and Biodiversity.

[ref3] (2012). Chemical composition and biological activities of the essential oil of Laggera aurita Linn (DC.) grown in Pakistan. Turkish Journal of Biochemistry.

[ref4] (Giles 1902). Effect of essential oils of six local plants used insecticide on adults of Anopheles gambiae. Journal of Entomology.

[ref5] (2016). Isolation, structure elucidation and larvicidal activity of Laggera alata extracts. Research Journal of Pharmacognosy and Phytochemistry.

[ref6] (1999). Phytotoxins from the leaves of Laggera decurrens. Journal of Agricultural and Food Chemistry.

[ref7] (2010). Chemical constituents of Laggera pterodonta. China Journal of Chinese Materia Medica.

[ref8] (1S). -one, from the essential oil of the Ethiopian plant Laggera tomentosa. Trimethylbicyclo [3.1.

[ref9] (2013). Ethno-medicinal study of plants used for treatment of human and livestock ailments by traditional healers in South Omo, Southern Ethiopia. Journal of Ethnobiology and Ethnomedicine.

[ref10] (2003). Constituents of the essential oil of Laggera tomentosa Sch. Bip. ex Oliv.

[ref11] (1995). Identification of Essential Oil Components by GC-MS.

[ref12] (2017). Chemical composition of Mentha pulegium and Rosmarinus officinalis essential oils and their antileishmanial, antibacterial and antioxidant activities. Microbial Pathogenesis.

[ref13] (2019). Phytochemical composition and bioactivities of essential oils from six Lamiaceae species. Industrial Crops and Products.

[ref14] (2011). Juniperus sibirica Burgsdorf. as a novel source of antioxidant and anti-inflammatory agents. Food Chemistry.

[ref15] (2010). Antioxidant properties of resveratrol: a structure–activity insight. Innovative Food Science and Emerging Technologies.

[ref16] (2007). Antioxidant and antimicrobial activities of shiitake (Lentinula edodes) extracts obtained by organic solvents and supercritical fluids. Journal of Food Engineering.

[ref17] (2013). Antimicrobial activity and chemical composition of the essential oils of mosses (Hylocomium splendens (Hedw.) Schimp. and Leucodon sciuroides (Hedw. Turkish Journal of Chemistry.

[ref18] (2006). Composition and antimicrobial activities of volatile components of Minuartia meyeri. Turkish Journal of Chemistry.

[ref19] (1990). -Hydroxycarvotanacetone and other constituents of the essential oil of Laggera alata (D. Don) Sch. Flavour and Fragrance Journal.

[ref20] Composition of the essential oil of Laggera alata. Planta.

[ref21] (2002). Composition of the essential oils from three Laggera spp. from Cameroon. Flavour and Fragrance Journal.

[ref22] (2000). Investigation of the essential oil and headspace of Laggera pterodonta (DC.) Sch. Bip. ex Oliv., a medicinal plant from Cameroon. Journal of Essential Oil Research.

[ref23] (2014). Chemical composition and antimicrobial activities of essential oils of Laggera pterodonta collected in four different locations in Yunnan, China. Plant Diversity.

[ref24] (2019). Characterization of a new epoxy-hydroxycarvotanacetone derivative from the leaf essential oil of Laggera pterodonta from Côte d’Ivoire. Natural Product Research.

[ref25] (2006). Chemical composition of the volatile oil of Laggera aurita Schulz from Burkina-Faso. Biochemical Systematics and Ecology.

[ref26] (2015). Chemical composition and antibacterial activity of the essential oils of Laggera crispata (Vahl) Hepper & Wood, Cyclospermum leptophyllum (Pers.) Eichler and Perilla frutescens (L.) Britton. Analytical Chemistry Letters.

[ref27] (2011). Chemical investigation of the essential oil of Laggera crispata (Vahl) Hepper & Wood from India. Journal of the Serbian Chemical Society.

[ref28] (2013). Compositional variation in the essential oils of vegetative and reproductive parts of Laggera crispata (Vahl) Hepper & Wood. National Academy Science Letters.

[ref29] (2012). Evaluation of the antimicrobial and antioxidant activities of essential oils, extracts and their main components from oregano from Madeira Island.

[ref30] (2018). Chemical composition, antioxidant activity and antifungal effects of five Iranian essential oils against Candida strains isolated from urine samples. Journal de Mycologie Medicale.

[ref31] (2020). GC-MS analysis of the methanolic extracts of Smilax china and Salix alba and their antioxidant activity. Turkish Journal of Chemistry.

[ref32] (2011). Phytochemical analysis and in vitro antimicrobial and free radical-scavenging activities of the essential oils from Euryops arabicus and Laggera decurrens. Molecules.

[ref33] (2013). Chemical constituents and antibacterial property of the essential oil of the roots of Cyathocline purpurea. Journal of Ethnopharmacology.

[ref34] (2008). Biological effects of essential oils–a review. Food and Chemical Toxicology.

[ref35] (2014). Composition and activity against oral pathogens of the essential oil of Melampodium divaricatum (Rich. Chemistry and Biodiversity.

[ref36] (2020). Chemical composition and biological activity of essential oils from Aloe debrana roots. Journal of Essential Oil Bearing Plants.

[ref37] (2006). GC-MS analysis and antibacterial activity of cultivated Satureja cuneifolia Ten. essential oil. Turkish Journal of Chemistry.

[ref38] (2020). Chemical composition, antibacterial and antioxidant activities of oils obtained by different extraction methods from Lepidium sativum L. seeds. Industrial Crops and Products.

